# Thrombose veineuse cérébrale de l’enfant: à propos d’une série de 12 cas

**DOI:** 10.11604/pamj.2019.32.22.17656

**Published:** 2019-01-15

**Authors:** Naima Baddouh, Safaa Elbakri, Ghizlane Draiss, Youssef Mouaffak, Noureddine Rada, Said Younous, Mohammed Bouskraoui

**Affiliations:** 1 Service de Pédiatrie A, CHU Med VI Marrakech, Faculté de Médecine et de Pharmacie, Université, Cadi Ayyad, Marrakech, Maroc; 2 Service de Réanimation Pédiatrique, CHU Med VI Marrakech, Faculté de Médecine et de Pharmacie, Université, Cadi Ayyad, Marrakech, Maroc

**Keywords:** Anti thrombotique, enfant, imagerie, thrombose veineuse cérébrale, Antithrombotic, child, imaging, cerebral venous thrombosis

## Abstract

La thrombose veineuse cérébrale (TVC) est rare chez l'enfant. Sa présentation clinique et ses étiologies sont variables. Le pronostic des patients reste redoutable devant le risque de décès et des séquelles neurosensorielles. L'objectif était d'étudier le profil clinique, radiologique et étiologique des TVC de l'enfant, et évaluer l'intérêt du traitement anti thrombotique. L'étude rétrospective menée au service de pédiatrie et de réanimation pédiatrique au CHU Med VI de Marrakech, Maroc, sur une période de 9 ans 10 mois (janvier 2008 à octobre 2018), colligeant tous les cas de TVC confirmés à l'imagerie, ayant un âge entre 1 mois et 15 ans. Nous avons recensé 12 cas. L'âge moyen était de 6,4 ans. Le sexe ratio était de 1.4. Le mode de début était aigu dans 7 cas. Les principales présentations cliniques étaient les convulsions (7 cas), les signes neurologiques focaux (7 cas), et les signes d'hypertension intracrânienne (HTIC) (6 cas). La tomodensitométrie (TDM) et / ou l'imagerie par résonance magnétique (IRM), ont révélé une atteinte du réseau veineux superficiel dans 8 cas, étendue dans 3 cas. L'étiologie était infectieuse chez 6 patients avec un cas de déshydratation, deux cas de maladie de système et une homocystinurie. Cependant, l'étiologie restait inconnue chez deux patients. Le traitement anti-thrombotique instaurait chez 7 enfants, avait permis une bonne évolution clinico-radiologique dans 5 cas. Le décès était survenu chez 2 enfants, et 3 autres avaient des séquelles neurologiques. Les TVCs chez l'enfant sont caractérisées par la grande diversité de leur présentation clinique et de leurs étiologies. L'impact des anticoagulants a été prouvé malgré l'absence de protocole thérapeutique standardisé.

## Abstract (en)

Cerebral venous thrombosis (CVT) is rare in children. Its clinical features and its cause vary. Prognosis is dreadful due to the risk of death and neurosensory sequelae. This study aims to examine the clinical, radiological and etiological profile of CVTs in children and to evaluate the role of antithrombotic treatment. We conducted a retrospective study in the Department of Paediatrics and Paediatric Resuscitation at the Center Hospital University Mohammad VI (CHU) in Marrakech, Morocco, over a period of nine years and ten months (January 2008-October 2018). We collected data from the medical records of all patients aged between 1 months and 15 years with CVT confirmed by imaging. We listed 12 cases of CVT. The average age of patients was 6.4 years. Sex ratio was 1.4. Acute onset occurred in 7 cases. The main clinical features of CVT included seizures (7 cases), focal neurologic signs (7 cases) and signs of intracranial hypertension (IH) (6 cases). CT scan and/or magnetic resonance imaging (MRI) revealed an involvement of the superficial venous network in 8 cases and extended venous involvement in 3 cases. In six cases CVT was caused by an infection, with a case of dehydration, two cases of systemic disease and a case of homocystinuria. However, the cause of the disease was unknown in two patients. Seven children were treated with antithrombotic therapy with good clinico-radiological outcome in 5 cases. Two children died and 3 others had neurological sequelae. In children, CVTs are characterized by a vast variety of clinical features and causes. The effect of anticoagulant therapy was demonstrated despite the absence of a standardized therapeutic protocol.

## Introduction

La thrombose veineuse cérébrale (TVC) est une pathologie rare particulièrement chez l'enfant. Son incidence varie entre 0,4 et 0,7 pour 100.000 enfants par an [1]. La symptomatologie clinique est non spécifique, et le diagnostic est souvent retardé. Néanmoins, elle est de plus en plus diagnostiquée grâce à la sensibilisation des cliniciens et aux progrès actuels en neuro-imagerie [2]. La morbi-mortalité reste élevée chez l'enfant vu la survie prolongée de l'enfant après l'incident comparativement à l'adulte [3]. Les consensus actuels recommandent l'utilisation des anticoagulants pour le traitement des TVC de l'enfant [4]. Peu d'études se sont intéressées aux TVC de l'enfant, particulièrement en Afrique [5]. Nous avons ainsi réalisé une étude rétrospective d'une série de TVC de l'enfant colligée, aux services de pédiatrie et de réanimation pédiatrique du CHU Med VI Marrakech, Maroc, sur une période de 9 ans 10 mois afin de déterminer les caractéristiques cliniques, radiologiques, étiologiques et évolutifs ainsi que les modalités de prise en charge de cette affection dans cette tranche d'âge dans un pays africain.

## Méthodes

Il s'agit d'une étude rétrospective sur les cas de TVC, pris en charge au service de pédiatrie et de réanimation pédiatrique de l´hôpital Mohammed VI à Marrakech, durant la période 2008-2018. Le diagnostic de la TVC était établi sur les données du scanner cérébral (sans et avec injection de produit de contraste) et l'imagerie par résonance magnétique (IRM) associée à des séquences angiographiques. Plusieurs paramètres étaient étudiés: l'âge, le sexe, les antécédents de thrombophilie personnels ou familiaux, la symptomatologie initiale et le mode de survenue (aiguë < 2jours, subaiguë: 2jours à 1mois, chronique > 1mois), les caractéristiques radiologiques et la topographie des lésions. La localisation de la thrombose était classée comme superficielle ou profonde, unique ou étendue, et les lésions parenchymateuses étaient recherchées systématiquement. L'enquête étiologique incluait systématiquement un bilan infectieux complet, un examen oto-rhino-laryngologique (ORL) et ophtalmologique, l'étude de l'hémostase, les D-dimères, l'homocystéine, le dosage des protéines C et S, de l'antithrombine III, le dosage du facteur V Leiden et d'autres bilan en fonction de l'orientation étiologique. Les modalités de prise en charge et le pronostic fonctionnel et vital ont été évalués aussi. L'analyse a été faite à l'aide des statistiques descriptives sur EXCEL.

## Résultats

**Figure 1:**
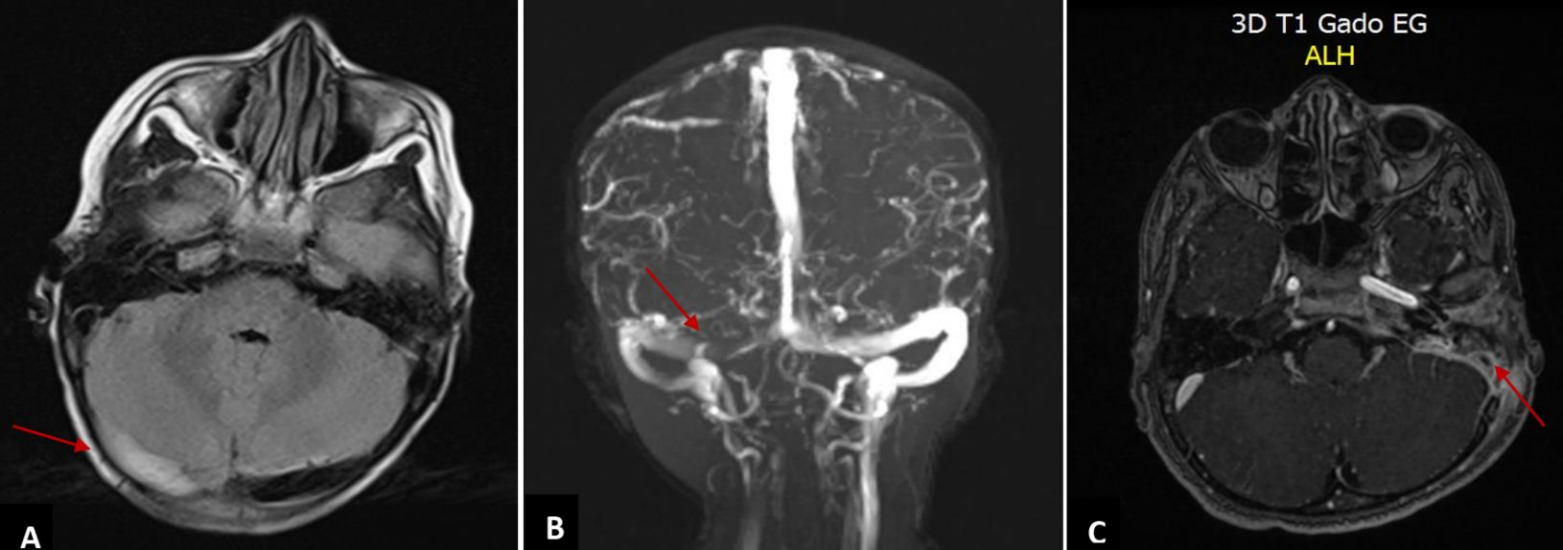
Images illustrant l’aspect d’une thrombose veineuse cérébrale sur l’IRM cérébrale chez nos patients; A et B) IRM cérébrale et angio IRM montrant l’aspect d’une thrombose veineuse du sinus latéral droit; C) thrombose veineuse du sinus sigmoïde gauche visualisée sur une coupe axiale d’une IRM cérébrale

**Figure 2:**
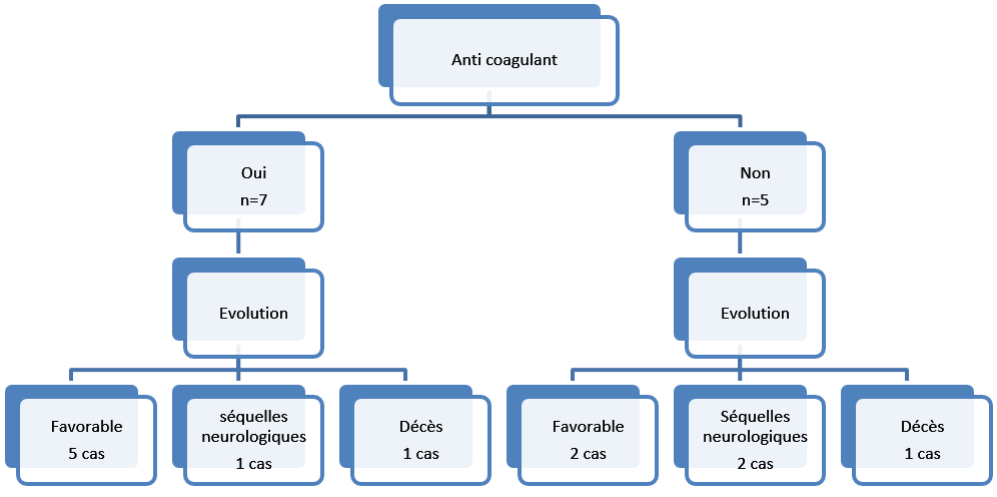
Impact du traitement anticoagulant sur l’évolution des patients

Nous avons recensé 12 cas. L'âge moyen des patients était de 6,4 ans (extrêmes de 50 jrs à 13 ans). Le sexe masculin était prédominant avec un sexe ratio à 1,4. Le mode d'installation des TVC était aigu chez 7 patients et subaigu chez 6. Le tableau clinique était variable, et les principales présentations révélatrices étaient : les crises convulsives, les signes neurologiques diffus et/ou focaux, et les signes d'hypertension intracrânienne (HTIC) (Tableau 1). Le scanner cérébrale était l'examen de 1ère intention, demandé en condition d'urgence (à défaut de la disponibilité de l'IRM). Il a permis de visualiser les signes directs et indirects de la TVC dans 8 cas. L'IRM cérébrale avec séquence ARM réalisée chez 8 patients, avait permis de confirmer le thrombus veineux dans 3 cas (TDM non concluante) et sa topographie dans 5 cas intéressant par ordre de fréquence décroissant: le sinus latéral (Figure 1), le sinus longitudinal, le sinus sigmoïde (Figure 1) et le sinus sagittal. Des thromboses multiples et profondes concernant à la fois les sinus et les veines cérébrales, avaient été observées chez 2 enfants. Des lésions parenchymateuses cérébrales associées avaient été objectivées dans 2 cas. Il s'agissait d'un ramollissement hémorragique. Les causes infectieuses représentaient l'étiologie de la TVC la plus fréquemment retrouvée dans notre série (6 patients). Il s'agissait de cause locale dans 5 cas (otite, otomastoidite, méningite), et systémique dans 1 des cas (Tableau 1). L'enquête étiologique avait permis de diagnostiquer chez les autres patients un cas de déshydratation, un cas d'homocystinurie et deux cas de maladie de système: un cas de maladie de Behçet et un autre cas dont le bilan de confirmation est en cours, alors qu'aucune étiologie n'a été trouvée chez 2 enfants. Le bilan de la thrombophilie fait chez 8 patients s'est révélé négative. Tous les malades étaient mis initialement sous antibiothérapie probabiliste, avant d'éliminer une thrombose septique. Le traitement anticoagulant a été instauré chez 7 enfants. Il était à base d'héparine de bas poids moléculaire à la phase aiguë, puis, un anti vitamine K, durant une période allant de 3 à 6 mois. D'autres thérapies ont été utilisées selon la présentation clinique et étiologique (antalgique, anticonvulsivant, antibiotique, Réhydratation, corticothérapie). L'évolution clinique et radiologique était favorable avec récupération complète dans 7 cas, dont 5 parmi eux avaient reçu des anticoagulants (Figure 2). Des séquelles neurologiques étaient observées chez 3 enfants: une atrophie optique, une paralysie du nerf VI, et une épilepsie. L'évolution était fatale chez 2 enfants, à cause d'une atteinte diffuse et profonde chez un enfant ayant une homocystinurie, et un tableau de déshydratation sévère avec insuffisance rénale organique chez l'autre.

**Tableau 1:** Les données démographiques, cliniques et para cliniques des patients

Nombre des cas	âge	sexe	Signes cliniques	localisation	Bilan de thrombophilie	Etiologie
1	7 ans	F	HTIC Diplopie binoculaire	Sinus sigmoïde droit	Non fait	Infection ORL
2	13 ans	M	HTIC Baisse de l’A.V	Sinus latéral et Sigmoïde	Sans anomalie	Maladie de Behҫet
3	6 ans	M	Etat de mal convulsif	Sinus latéral droit	Non fait	Non retrouvée
4	12 ans	F	HTIC EMC partiel Hémiparésie	Sinus longitudinal supérieur	Sans anomalie	Infection systémique
5	50 jours	M	Trouble de conscience Hypotonie	Multiple : Sinus latéral / Sinus longitudinal /sigmoïde/sinus droit	Non fait	Déshydratation
6	5 mois	M	EMC partiel Hémiparésie	Sinus longitudinal supérieur	Non fait	Non retrouvée
7	2 mois	F	EMC partiel	Sinus longitudinal supérieur	Sans anomalie	Infection neuro méningée
8	7 ans	F	Sd d’HTIC Œdème papillaire	Sinus longitudinal supérieur	Sans anomalie	Maladie de système
9	20 mois	M	Convulsions	Sinus latéral	Sans anomalie	Otomastoidite
10	6 ans	F	Convulsions hémiplégie	Sinus sigmoïde gauche	Sans anomalie	Méningite
11	9 ans	M	Trouble de conscience HTIC Tétra parésie	Sinus latéral	Sans anomalie	Homocystinurie
12	10	M	masse rétro auriculaire gauche otorrhée fétide paralysie faciale périphérique	Sinus sigmoïde gauche	Sans anomalie	Otomastoidite

## Discussion

L'incidence annuelle de la TVC reste sous-estimée du faite de l´anatomie variable et la recanalisation rapide des canaux sino-veineux, le polymorphisme clinique, et l'utilisation des anciennes techniques d'imagerie [6]. En Afrique, très peu de cas ont été décrits [5], nous présentant une série de 12 cas colligée sur une durée de presque 10 ans, ce qui prouve que c'est une pathologie relativement rare dans notre contexte. La plupart des études ont inclus le nouveau-né chez qui l'incidence de TVC est plus élevée par rapport à l'enfant (2,6-12 pour 100.000 nouveau-nés par an) [7]. Nous avons exclu le nouveau-né dans notre étude. Les enfants ayant un âge > 2 ans représentaient 72% dans notre série, avec un moyen d'âge de 6,4 ans. Conformément à ce qui a été rapporté dans la littérature le sexe masculin prédomine dans notre série (sexe ratio: 1,4). La TVC chez l'enfant est caractérisée par son polymorphisme clinique. Les convulsions, les céphalées et les troubles de conscience représentent les signes les plus fréquemment rapportés dans les études [1]. Les convulsions, les déficits focaux et les troubles de conscience sont les signes cliniques les plus rapportés chez nos patients. L'angio IRM est l'examen de référence pour le diagnostic et le suivi des TVC [8]. Dans notre contexte, le scanner cérébral est l'imagerie accessible en condition d'urgence, réalisé chez tous nos patients; il n'était concluant que chez 8 enfants. L'IRM effectuée chez 7 enfants, a permis de confirmer le diagnostic en visualisant la topographie des TVC dans 3 cas, et des anomalies parenchymateuses associées chez 2 malades. Dans la littérature comme dans notre population, les sinus latéraux et le sinus longitudinal supérieur est le siège de prédilection des thrombophlébites cérébrales [9]. La TVC chez l'enfant est une affection multifactorielle, les étiologies sont multiples. Les causes générales sont dominées par les infections, la déshydratation, les anémies, à côté des pathologies chroniques prédisposantes aux TVC tel le syndrome néphrotique, les cardiopathies congénitales et le lupus érythémateux disséminé [10]. Toutefois, La TVC chez l'enfant est associé souvent à une pathologie locale de la tête et du cou, y compris les traumatismes crâniens, les tumeurs du système nerveux central ou chirurgie intracrânienne récente [11]. Historiquement, la TVC était une complication bien connue de l´otite moyenne aiguë et la mastoïdite. Elle a été identifiée dans 24% à 62% des cas de toutes les séries publiées au cours de la dernière décennie [12]. En effet avec l'avènement des antibiotiques, les causes infectieuses sont de plus en plus rares dans les pays occidentaux cependant elles prédominent dans les pays en voie de développement [5, 13], comme c'est le cas dans notre étude (la moitié des cas).

Dans l'enquête étiologique d'une thrombose veineuse cérébrale chez l'enfant, la recherche d'un facteur de risque thrombotique congénital ou acquis est systématique. Des états pro thrombotiques ont été rapportés dans 24% à 64% des séries d'enfants. Cependant, ces données sont difficiles à interpréter vu l'apparition récemment de nouveaux tests et leur variabilité en fonction des enfants et du moment du test [11, 12, 14, 15]. Vu le manque de réactif ou de moyens, nous n'avons pas pu réaliser le bilan de thrombophilie que chez huit de nos patients, et il est revenu négative, tout en sachant que le bilan disponible dans notre formation ne comporte que la protéine C, S, le facteur V et l'antithrombine III. Les deux cas de maladie de système et d'homocystinurie retrouvés dans notre série sont considérés comme cause rare de la TVC [16, 17]. Même avec un bilan exhaustif, 20 à 25% des étiologies des TVC restent indéterminées [18]. Aucune étiologie n'est retrouvée chez deux de nos patients. Malgré l'absence des études randomisées contrôlées, les anticoagulants restent largement utilisés chez l'enfant, ils permettent d'améliorer le pronostic vital et fonctionnel, et diminuer le risque de récidive [4, 19, 20]. Nous avons utilisé chez nos patients l'énoxaparine sodé en raison de sa bonne biodisponibilité, sa demi-vie plus longue et le risque moindre de saignement [21]. Nos patients ont été traités par héparine, sauf ceux qui ont eu des lésions hémorragiques. La durée moyenne de traitement habituellement retenue chez l'enfant est de 3 à 6 mois [10]. Tous nos patients étaient traités aussi pendant une durée de 3 à 6 mois. Nous avons constaté dans notre série une évolution favorable chez 5/7 des enfants traités par anticoagulant contre 2/5 des enfants non traités. Ces résultats peuvent plaider en faveur de l'efficacité des anticoagulants malgré la petite taille de notre échantillon. L'évolution clinique et radiologique était favorable avec récupération complète dans 7 cas, dont 5 parmi eux avaient reçu des anticoagulants. Le pronostic des TVC dépend essentiellement de l'âge de l'enfant, l'étiologie et la localisation de la TVC [10, 22]. Egalement dans notre étude, l'évolution était fatale chez 2 enfants, à cause d'une atteinte diffuse et profonde chez un enfant ayant une homocystinurie, et un tableau de déshydratation sévère avec insuffisance rénale organique chez l'autre. La mortalité spécifique par TVC est inférieure à 10%, mais les déficits neurologiques résiduels sont rapportés dans entre 17% et 79% des cas [7, 12, 23, 24]. Des séquelles irréversibles ont été notées chez 27% de nos enfants avec un taux de mortalité de 18 %. Le taux de séquelles neurologiques résiduelles chez nos patients est inférieur à celui rapporté dans la plupart des études [3, 25].

## Conclusion

La TVC chez l'enfant représente un challenge diagnostique et thérapeutique vu son polymorphisme clinique et l'absence d'un consensus international de prise en charge. Les causes infectieuses représentent l'étiologie la plus fréquemment retrouvée chez nos patients d'où l'intérêt d'un traitement précoce et adéquat des infections ORL et cérébro méningée dans la prévention des TVC dans notre contexte. Malgré qu'il ne soit pas codifié, Le traitement par anticoagulant a démontré son efficacité dans la réduction de la morbi-mortalité associée aux TVC chez l'enfant. Plus d'études prospectives randomisées et contrôlées s'avèrent nécessaires pour codifier ce traitement dans la population pédiatrique.

### Etat des connaissances actuelles sur le sujet

- Pathologie rare chez l'enfant ;
- Peu de séries de cas publiées chez l'enfant particulièrement en Afrique;
- L'utilisation des anticoagulants dans le traitement de la thrombose veineuse cérébrale de l'enfant est sujette de controverse.

### Contribution de notre étude à la connaissance

- Une série de cas colligée dans un pays africain;
- La bonne évolution chez les patients mis sous traitement anticoagulant par rapport à ceux chez qui ce traitement n'a pas été instauré;
- La fréquence des causes infectieuses d'où la possibilité de prévention de cette pathologie dans notre contexte via la prise en charge des infections ORL et cérébro-méningées.

## Conflits des intérêts

Les auteurs déclarent ne pas avoir de conflits d'intérêts.
